# Globalization and the rise and fall of cognitive control

**DOI:** 10.1038/s41467-020-16850-0

**Published:** 2020-06-18

**Authors:** Mohsen Mosleh, Katelynn Kyker, Jonathan D. Cohen, David G. Rand

**Affiliations:** 1 Sloan School of Management, Massachusetts Institute of Technology, Cambridge, MA 02138 USA; 2 Department of Psychology, Yale University, New Haven, CT 06520 USA; 3 Princeton Neuroscience Institute, Princeton, NJ 08540 USA; 4 Department of Psychology, Princeton, NJ 08540 USA; 5 Institute for Data, Systems, and Society, Massachusetts Institute of Technology, Cambridge, MA 02138 USA; 6 Department of Brain and Cognitive Sciences, Massachusetts Institute of Technology, Cambridge, MA 02138 USA

**Keywords:** Cultural evolution, Human behaviour

## Abstract

The scale of human interaction is larger than ever before—people regularly interact with and learn from others around the world, and everyone impacts the global environment. We develop an evolutionary game theory model to ask how the scale of interaction affects the evolution of cognition. Our agents make decisions using automatic (e.g., reflexive) versus controlled (e.g., deliberative) cognition, interact with each other, and influence the environment (i.e., game payoffs). We find that globalized direct contact between agents can either favor or disfavor control, depending on whether controlled agents are harmed or helped by contact with automatic agents; globalized environment disfavors cognitive control, while also promoting strategic diversity and fostering mesoscale communities of more versus less controlled agents; and globalized learning destroys mesoscale communities and homogenizes the population. These results emphasize the importance of the scale of interaction for the evolution of cognition, and help shed light on modern challenges.

## Abstract (web summary)

Humankind is in a period of unprecedented cognitive sophistication as well as globalization. Here, using an evolutionary game theory model, the authors reveal ways in which the transition from local to global interaction can have both positive and potentially negative consequences for the prevalence of cognitive sophistication in the population.

## Introduction

Humanity is experiencing a period of unprecedented innovation and technological sophistication, as well as unprecedented interconnectedness and globalization. However, many of these innovations and technological advancements are often misused in a short-sighted fashion (e.g., the over-prescription of antibiotics, abuse of opiates, and over-reliance on fossil fuels^1^). In parallel, a substantial backlash is occurring against the kind of expertise and logical, deliberative thinking that produced such innovations (e.g., an increasing rejection of scientific expertise^2^ and disregard for the factualness of news^3^). In this paper, we examine how the uniquely interconnected state of the modern world might influence these societal dynamics.

We do so by combining two traditionally distinct theoretical perspectives^4–6^: cognitive psychology and evolutionary dynamics. With respect to cognition, we focus on a critical dimension of cognitive function that spans from automatic to controlled processing^7–10^. Along this dimension, automatic cognitive processes are more hardwired or over-learned habits, which leads to greater efficiency (e.g., greater speed and less effort) when they are appropriate, but at the cost of reduced flexibility (e.g., the ability to quickly adjust to the details of the current situation). More controlled processes, conversely, involve more deliberation and thought—requiring greater investment of time and effort, but more quickly responding to changes of circumstance and/or the specifics of a particular decision. Despite the immense body of work on automatic versus controlled processing, this distinction has been almost entirely absent from evolutionary game theory until recently^11^. Because humans do not exist in isolation, it is essential to consider population-level dynamics (e.g., the interaction between different agents) and the impact that cognition has on the environment. Evolutionary game theory provides the ideal tool for formally modeling such effects. For example, in the context of cooperation^12–15^, models have demonstrated the evolution of intuitive cooperation and deliberative self-interest. And in the context of cognition-environment feedback effects^4–6^, models have demonstrated the emergence of persistent cycles of automatic versus controlled processing.

Here, we integrate this combination of cognitive psychological^16–19^ and evolutionary game theoretic approaches with social network models^20–32^ to explore how the evolution of cognitive control is influenced by local versus global interaction structures. We simulate the evolutionary dynamics of a population of agents embedded in a network who vary along the dimension of automatic versus controlled processing (as defined below). Specifically, we add population structure to a multi-process agent model^4^ in which agent *i* engages in automatic processing with probability 1*-x*_*i*_ and controlled processing with probability *x*_*i*_. As described in detail below, agents earn fitnesses based on their strategy, the strategy of other agents in the population, and the state of the environment; and then an evolutionary process occurs in which agents with lower fitness tend to adopt the strategies of agents with higher fitness (or die and be replaced with offspring of higher fitness agents).

Specifically, the fitness of agent *i* in timestep *t* is given by:

1 $$f_{i,t} = x_i\pi _t^C + (1 - x_i)\pi _t^A$$

where $\pi _t^C$ is the payoff of engaging in controlled processing and $\pi _t^A$ is the payoff of engaging in automatic processing. These terms are then defined as:

2 $$\pi_{{t}}^{{C}} = 1 - {{c}} + {{w}}(1 - < {{x}} > _{{t}})$$

3 $$\pi _{{t}}^{{A}} = 1 - {{p}}_{{t}}$$

4 $${{p}}_{{t}} = 1 - < {{x}} > _{{{t}} - 1}$$

where:

*c* is the fixed cost of controlled processing.

*w* is the impact of automatic processing on the cost of controlled processing.

*<x>*_*t*_ is the average probability of controlled processing in the population at time *t*.

*p*_*t*_ is the state of the environment at time *t*.

We now explain the logic behind these definitions. Our modeling of the payoff consequences of automatic versus controlled processing ($\pi _t^C$ and $\pi _t^A$) are based on the trade-off between efficiency versus flexibility of cognitive processing. One of the central tenets of cognitive psychology and neuroscience is that the defining feature of control-dependent processing (relative to automatic) is slower but more flexible responding; that is, the ability to respond to a stimulus more slowly but in a non-habitual, contextually-relevant way. Automatic processing is therefore conceptualized as supporting efficient and typically effective behavior, achieved by encoding pre-compiled responses that are optimally adapted to a particular set of circumstances, but are slow to develop or adapt in response to environmental change or experience. In contrast, controlled processing is conceptualized as supporting a more flexible form of processing that can adjust more quickly to changes in contingencies and thereby generate advantageous responses under a wider range of conditions. Critically, however, this flexibility comes at a cost^33–38^.

The distinction between automatic and controlled processing that we are capturing in our model can be seen in a wide range of everyday behaviors. While automatic processing is critical to the efficient and often skillful functioning of an agent (e.g., most of our physiological regulatory processes are automatic, and the development of automaticity is fundamental to the acquisition of complex skills, such as typing, playing the piano, driving a car, etc.), nevertheless, automatic behavior can be disadvantageous in many circumstances, especially when its immediate benefits are outweighed by longer term negative consequences. For example, research shows that automatic processing favors immediate gratification whereas the exercise of control promotes delayed gratification; and that controlled processing facilitates planning for the future. Accordingly, reliance on automatic processing can be associated with making poor investment decisions^39^, being overweight^40^, using addictive substances like alcohol, tobacco, and illegal drugs^41^, and preferring simple over nuanced information sources^42^, among many other things. Controlled processing is also central to the reasoning and analytic thinking that enable humans to solve complex problems, to be creative, and to achieve scientific and technological advances^43^.

Across all of these examples of day-to-day decision-making, the common thread is that controlled processing can often lead to better decision-making outcomes, but is more costly (e.g., requires more time and mental effort). To capture the effect of this relationship on average in our model, the basic decision-making payoff of automatic processing (1−*p*_*t*_ with 0 < *p*_*t*_ < 1) is less than the basic decision-making payoff of control (1). Thus *p*_*t*_ describes the relative advantage of controlled decision-making in time period *t*. In addition to these payoffs from decision-making, $\pi _t^C$ expression also includes the fixed cost *c* paid to engage in controlling processing.

Our basic payoff specification also includes a social element, captured by the *w*(1− <*x*>_*t*_) term of $\pi _t^C$. This describes the payoff that controlled agents receive from engaging with automatic agents. The parameter *w* can either be negative (such that interacting with agents acting automatically reduces the payoff of controlled processing) or positive (such that interacting with agents acting automatically increases the payoff of controlled processing). Negative *w* reflects situations in which interacting with automatic agents reduces the payoff of controlled agents. For example, because automatic processing is faster than controlled processing, in circumstances for which timing is a factor, an agent relying on automaticity may outcompete one relying on controlled-processing to acquire resources by acting immediately to acquire the resource while the controlled agent is more carefully considering its best course of action^5^. Alternatively, agents acting automatically can derail the plans of controlled decision-makers due to automaticity’s own lack of planning (e.g., irresponsible use of the externalities generated by controlled processing, such as abuse of antibiotics or reckless use of weaponry). Conversely, positive *w* reflects situations in which controlled agents benefit from interaction with automatic agents. For example, controlled decision-makers may profit by producing products that agents acting automatically preferentially desire (e.g., alcohol, drugs, and low quality news), or by exploiting the short-sightedness of agents acting automatically (e.g., manipulating automatic agents into false beliefs that benefit the controlled agent).

Finally, our modeling of the dynamics of *p* are designed to capture how the relative proportion of the two cognitive processing employed by agents in the population interacts—typically at much longer timescales than the day-to-day decision-making described above—with the environment. Specifically, we assume that the reasoning and analytic thinking enabled by controlled processing produce innovations and corresponding externalities that lead to a more hospitable environment, such as technologies that make the environment less variable and/or higher yield. This in turns makes the future more stable, reduces the importance of careful planning, and thus reduces the payoff advantage of controlled processing *p*. That is, the externalities generated by controlled processing help automatics as much as controls (without the automatics having to be the cost of control) because the externalities make it less necessary to exercise control in order to achieve good outcomes (i.e., controls and automatics both arrive at the same, better, outcome, but automatics do it without having to pay cost). For example, the invention of sophisticated irrigation and farming techniques makes food production much less susceptible to environmental variation, and in turn reduces the need to plan for the future by stockpiling against periods of drought. Or, in terms of the social environment, social science discoveries regarding the power of defaults have enabled policy makers to design institutions in which the optimal choice is the default (e.g., making saving for retirement opt-out rather than opt-in)—such that automatic agents are more likely to make optimal choices without having to exert control. Importantly, controlled processing is often necessary for maintaining these advances as well as developing them—without sufficient foresight and planning, and the willingness to invest in upkeep today in order to reap the benefit of functional systems tomorrow, the benefits of the technologies developed using controlled processing can be undermined.

We implement this cognition-environment feedback by having the relative advantage of controlled decision-making *p* decrease as the average level of controlled processing in the population *<x>* increases. Specifically, *p* in time period *t* is set equal to 1−<*x*> in period *t*−1, yielding the *p*_*t*_ expression given above. In a more complex version of the model, we also consider the impact of time lags on the interaction between the population and the environment—see “Methods” section for details.

We then study the population dynamics of automatic versus controlled processing using evolutionary game theory. Specifically, agents update their strategies using the death-birth Moran process with exponential fitness. In each generation, an agent (the learner) is randomly selected to update, and her probability of controlled processing is replaced with another agent (the teacher) selected proportional to an exponential function of the neighbors’ fitness; or, with probability *u*, a mutation occurs and the learner is assigned a new random strategy, sampled from a uniform distribution over the interval [0, 1].

Mathematically, this population dynamic could represent either genetic evolution or cultural evolution (i.e., social learning). In our interpretation of the results, we mostly focus on cultural evolution, which can occur over fairly rapid timescales. Individuals regularly update their strategies via social learning—that is, they compare their payoff to the payoffs of others, and (probabilistically) adopt the strategies of higher payoff others. To be consistent with standard convention in evolutionary game theory, we will refer to each strategy updating round as a “generation”, but remind the reader to not take this term too literally. That is, depending upon the processes and phenomena of interest, and how the model is applied, a “generation” could refer to updating at the cultural level as a consequence of social learning, or at the evolutionary time scale of reproduction.

Importantly, there are three different timescales within the model. The fastest timescale is the timescale of decision-making, which occurs on the timescale of minutes to hours. Each agent *i* makes many decisions every day—for example, decisions about whether to consume a resource or save it for the future, to eat an unhealthy meal or resist the temptation, to take a piece of information at face value or more carefully consider its credibility. The fraction of automatic versus controlled decisions is determined by the strategy parameter *x*_*i*_. Agents also interact with each other every day, with controlled agents incurring costs or gaining benefits from interacting with automatic agents, depending on the sign of *w*. Together, the payoffs from decisions and interactions on this decision making timescale determine the agent *i*’s fitness *f*_*i,t*_. Next is the timescale of strategy updating (i.e., learning), which is somewhat slower—e.g., on the order of months or years. Occasionally, agents compare their own success (i.e., payoff) with the success of other agents. If they find another agent that is regularly doing better than them, they (probabilistically) adopt that agent’s strategy. Finally, there is the timescale of cognition-environment feedback, whereby the level of control versus automaticity in the population affects the stability of the environment. With a sufficient investment of control-based analytic thinking, agents can invent and deploy new technologies that make life easier; and with sufficient lack of investment, these technologies can break down. These types of events tend to happen on a timescale that is longer than that of strategy updating. (Although this is less our focus, the model could also describe genetic evolution, in which the timescale of strategy updating—i.e., reproduction—would be longer, and more similar to the timescale of cognition-environment feedback.)

The main theoretical innovation of the present work is to add population structure to this model, in order to examine the impact of local versus global interactions on the evolutionary outcomes. For simplicity, our main analyses capture population structure by embedding agents in a ring-structured population in which they are connected to one neighbor on each side (results are not unique to the ring structure—for analysis of Small-world networks^44^ with varying strength of community structure, see Supplementary Information). We then examine the impact of local connectedness (restricted to neighbors) versus global connectedness (with all agents) on three different dimensions in our model: learning, contact between agents, and the environment.

With respect to learning (or reproduction), we vary how teachers are selected. In local learning the teacher is randomly selected from the immediate neighbors of the learner in the network. In global learning, the teacher is randomly selected from the whole population.

With respect to contact between agents, we vary how the fraction of controlled agents is calculated when determining the payoff consequences of contact between of agents with varying levels of automatic versus controlled processing (i.e., <*x*>_*t*_ in the *w*(1−<*x*>_*t*_) term in the payoff of controlled processing). In global contact, the fraction of controlled agents in the whole population is used to determine <*x*>_*t*_, as in the basic model formulation presented above. In local contact, conversely, the fraction of controlled neighbors is used —such that the fitness of controlled processing is impacted only by neighboring agents in the network. As a result, each agent *i* has its own fraction of controlled neighbors, such that <*x*>_*i*,*t*_ replaces <*x*>_*t*_ in the fitness function.

With respect to environment, we vary how the fraction of controlled agents is calculated when updating the environmental variable *p*_*t*_ that stipulates the decision-making advantage of controlled processing. In global environment, all agents experience the same value of *p*_*t*_, as in the main model formulation presented above. The value of *p*_*t*_ is then updated based on the average level of controlled processing across the entire population. In local environment, conversely, each agent *i* experiences its own local environment *p*_*i,t*_ which replaces *p*_*t*_ in the fitness function; and *p*_*i,t*_ is updated based on the average level of controlled processing of agent *i* and agent *i*’s immediate neighbors.

Together, these three different implementations of local versus global lead to eight (2^3^) possible combinations of local versus global connectedness, seven of which involve some element of globalization and one of which is entirely local. By examining model outcomes across these eight scenarios, we can explore the impact of differing forms of globalization on the temporal dynamics of how cognitive control evolves in the population.

## Results

### Globalization and average levels of control

We begin by examining the impact of different forms of globalization (i.e., of learning, contact, and/or environment) on the equilibrium average level of cognitive control within the population by comparing each of the seven globalization scenarios to the entirely local scenario.

Before directly examining the results of agent-based simulations, it is helpful to gain an intuition for the kinds of effects globalization has in the model, and why. To do so, we conduct an analysis using approximations of the fitness of automatic and controlled processing for a simplified case in which an agent is either fully controlled or fully automatic (see Supplementary Note 1). The results provide intuitions regarding the impact of each form of globalization. Globalization of the environment causes controlled agents to improve the environment experienced (and the payoff earned) by automatic agents throughout the population, and thus always results in lower levels of cognitive control in equilibrium. Globalization of contact increases the exposure of controlled agents to automatic agents, the consequences of which depend dramatically on *w*: this exposure decreases the payoff of control when automatic agents hurt controlled agents, *w* *<* 0, but increases the payoff of control when automatic agents benefit controlled agents, *w* *>* 0. Globalization of learning homogenizes the population and undercuts the clustering of strategies within the network, thereby weakening the effects of local contact and/or environment. Finally, all three forms of globalization interact, such that control is substantially higher in the fully local scenario compared to any of the globalization scenarios (provided that *w* *<* 0).

**Fig. 1:**
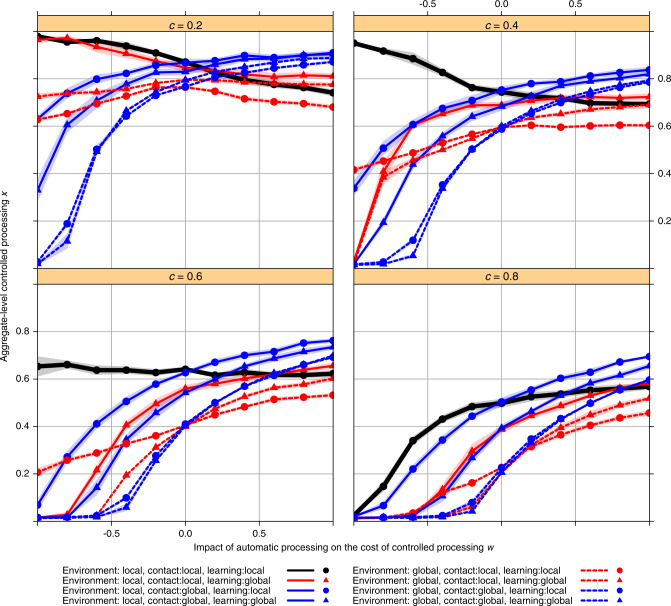
Aggregate-level controlled processing *x*. Each line represents a combination of local versus global contact, environment, and learning, across different levels of the impact of automatic processing on the cost of controlled processing (*w*) and fixed cost of controlled (*c*). Contact is indicated by color (red = local, blue = global). Environment is indicated by line type (solid = local, dashed = global). Learning is indicated by symbol (circle = local, triangle = global). All values are averaged over 10 simulation replicates. The shaded area represents the 95% confidence interval of <x> across simulation replicates for each set of parameters and combination of local versus global interaction.

We now present simulations of the full agent-based model. Agents were placed on a ring structure, and *c* and *w* were systematically varied across simulations. For each set of parameter values, we then simulated the model for 8 × 10^4^ generations and plotted the time-averaged frequency of control <*x*> averaged over 10 replicates for each set of simulation parameters (as shown in Supplementary Figs. 1 and 2, there is no significant variation across simulation replicates in the steady state value of <*x*>). In each simulation, we initialized the population from *x*_*i*_ = 0.01 (almost entirely automatic processing). Figure 1 shows that the simulation results generally accord with the predictions arising from the analytical approximation of the simplified system which are described above (and shown in Supplementary Table 1). When *w* is negative—such that the presence of automatic processing reduces the payoff of engaging in controlled processing—globalization of virtually any kind substantially reduces the average level of controlled processing in the population.

Conversely, when *w* is positive—such that the presence of automatic processing benefits those engaging in controlled processing—certain forms of globalization (specifically, global contact) can somewhat increase the average level of controlled processing in the population (although still maintaining a substantial level of automatic processing). Our results are robust to other network structures (see Supplementary Fig. 3) and larger networks (see Supplementary Fig. 4).

### Globalization and the dynamics of control

**Fig. 2:**
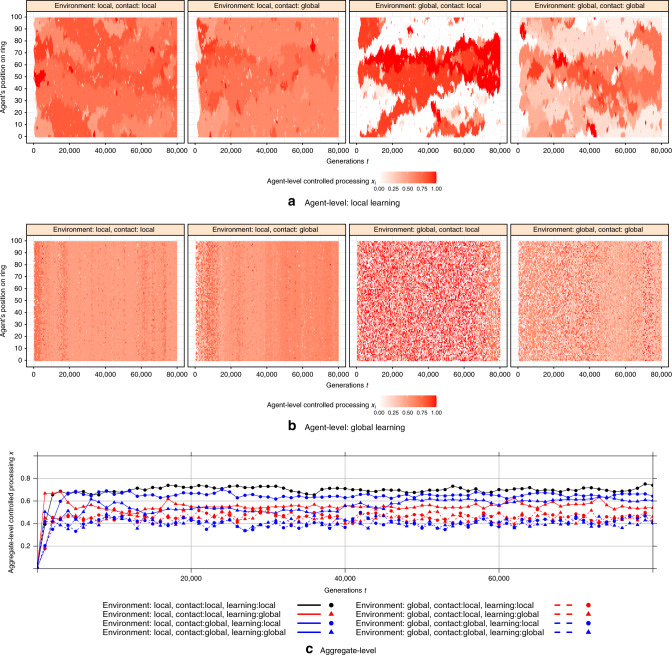
Spatiotemporal dynamics of automatic versus controlled processing. **a**, **b** Represent agent-level controlled processing *x* for each condition of localness over 8 × 10^4^ generations. Shown is the strategy of the agent in each location on the ring structure (on the *y* axis, such that pixels above and below each other are neighbors) at each generation (on the *x* axis), for each globalization scenario. **a** Shows results for global learning and panel (b) shows results for local learning. **c** Shows the aggregate-level controlled processing *x* over time for each condition of localness. Results are created for impact of automatic processing on the cost of controlled processing *w* = −0.15 and fixed cost of controlled *c* = 0.5.

The foregoing section addressed long-run average levels of control in the population. Here, we examine the impact of the scale of interaction on the dynamics of automaticity versus control, both across space and time (Fig. 2). We observe two striking effects.

The first concerns the emergence of mesoscale communities. Qualitatively, when learning is global (second row of Fig. 2), the entire population is well-mixed, and neither spatial nor temporal patterns are apparent. Conversely, as expected based on prior work on population structure^22^, when learning is local (top row of Fig. 2), communities of low versus high cognitive control emerge within the network (and the boundaries of these communities migrate over time). Interestingly, although global interactions tend to homogenize, we find a different pattern when it comes to the scale of environment: the emergence of mesoscale communities is markedly stronger when the environment is global rather than local. To quantify these differences in the extent of mesoscale communities, we calculate the average level of assortment *a* within the population (defined as the difference in (i) the fraction of an agent’s neighbors that have the same strategy as the agent versus (ii) the fraction of agents in the whole population—excluding the agent itself—that have the same strategy as the agent). Doing so shows that there is no assortment, *a* = 0, in any of the global learning scenarios – no mesoscale communities emerge. Conversely, there is substantial assortment when learning is local and environment is local (*a* = 0.65 for local contact, *a* = 0.65 for global contact); but even higher levels of assortment when learning is local but environment is global (*a* = 0.70 for local contact, *a* = 0.74 for global contact).

Why does local environment homogenize whereas global environment facilitates clusters? The answer arises from the particular anti-coordination payoff structure of automatic versus controlled agents in our model. When environment is local, the advantage of controlled processing *p*_*i,t*_ that each agent experiences is heavily influenced by that agent’s neighbors. If agent *i*’s neighbors are controlled, then *p*_*i,t*_ will be low and it will be advantageous for agent *i* to use automatic processing; if agent *i*’s neighbors are automatic, then the opposite will be true. Thus, it is more difficult to maintain clusters of agents with the same strategy—the local environment creates incentives to diverge from the strategies of one’s neighbors. Conversely, when the environment is global, all agents face the same *p*_*t*_, and thus the environment does not create an incentive to diverge from one’s neighbors.

The second striking feature in Fig. 2 involves the variance in *x* values across the population at any given time. Qualitatively, when the environment is local, there is relatively little variation in the level of control within the population at any given time—most agents show an intermediate level of control (pink coloration in Fig. 2). Conversely, when the environment is global, there is wide variation in the level of control within the population at any given time – some agents are almost entirely automatic (white) whereas others are almost entirely controlled (red). This is particularly true when contact is local. Quantitatively, the average value across time of the variance in *x* values in the population is very low when environment is local (0.000–0.008 depending on scale of contact and learning), higher when environment is global and contact is global (0.052 for local learning, 0.080 for global learning), and highest when environment is global and contact is local (0.171 for local learning, 0.181 for global learning).

The intuition for this variance-increasing effect of global environment is as follows. The basic structure of our model is such that the equilibrium involves either (*i*) a homogenous population in which all agents play a mixed strategy that randomizes over automatic and controlled processing, *x* *=* x* (leading to low variance across strategies) or (ii) a heterogeneous population in which fraction (1 − x*) of agents are totally automatic, *x* = 0, and fraction x* are totally controlled, *x* = 1 (leading to high variance across strategies). When environment is local, agents in different parts of the network face different values of *p* (and thus different selective forces on *x*) making it difficult to achieve the population-level coordination necessary for the heterogeneous solution. Conversely, when environment is global, all agents face the same value of *p*, and thus are better able to arrive at the heterogeneous solution. (For mesoscale measures on other network structures see Supplementary Fig. 5).

**Fig. 3:**
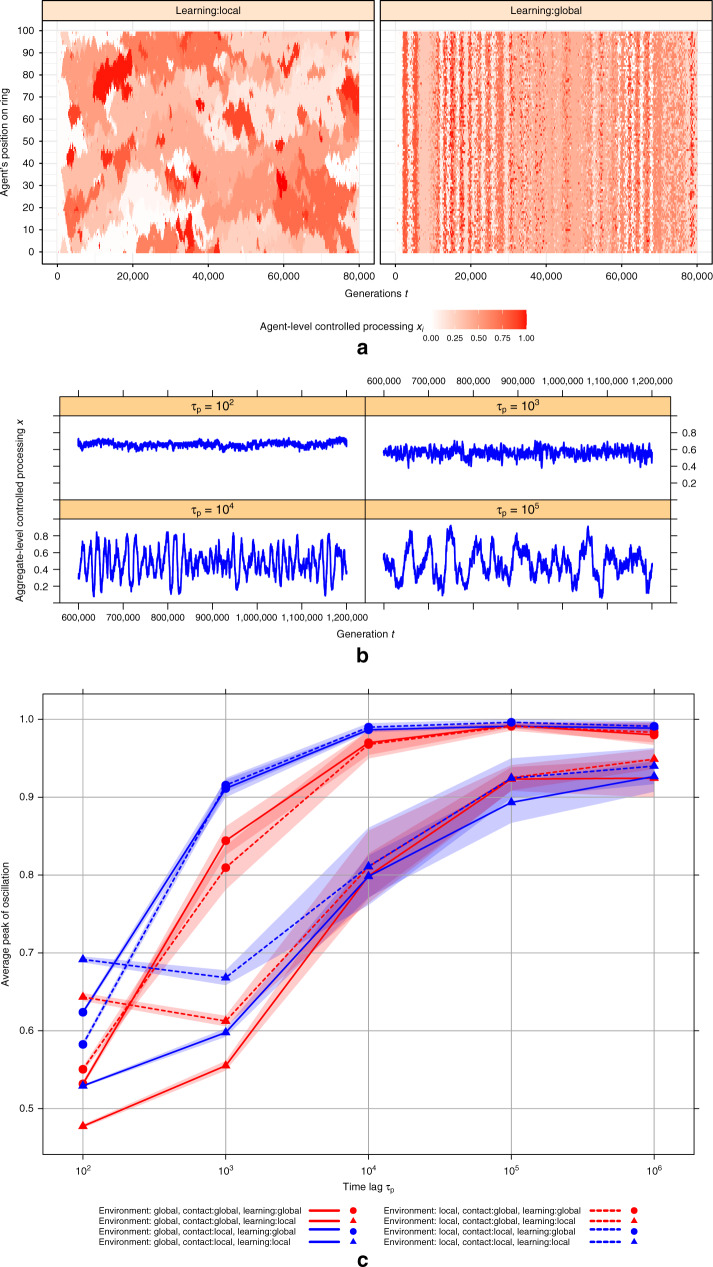
Effects of time lag *τ*_*p*_ on magnitude of oscillations. **a** Represents agent-level controlled processing *x* for global vs local learning (here time lag *τ*_*p*_ = 10^3^ and contact and environment are global, however, the spatial pattern does not qualitatively change for local contact and/or local environment). **b** Shows time series of oscillations of aggregate-level controlled processing *x* when contact, environment, and learning are local across different values of time lag. **c** Shows the average peak of oscillations for different condition of localness across different time lag. To cover all values of time delay, we run simulations over 1.2 × 10^6^ generations. Results are averaged over 10 simulation replicates. The shaded area represents the 95% confidence interval across simulation replicates for each set of parameter values and combination of local versus global interaction. Across all panels, the impact of automatic processing on the cost of controlled processing *w* = −0.15 and fixed cost of controlled *c* = 0.5.

Finally, we used the agent-based model to examine the more complex situation in which there is lag in the impact of cognition on the environment, parameterized by *τ*_*p*_ (see Methods for details of implementation). First, we examined the spatiotemporal distribution of strategies in the presence of lag for the situation in which all contact, environment, and learning are local versus global (Fig. 3a). Unlike the no-lag case considered in Fig. 2, strong temporal fluctuations are observed even in the fully globalized scenario. These fluctuations, although present, are less stark in the local scenario. When global, the population fluctuates between almost entirely automatic and entirely controlled (as has been shown previously in well-mixed populations^4^). When local, there is more coexistence of low and high control agents (due to the existence of local communities which are, to some extent, fluctuating out of phase with each other). Nonetheless, dramatic oscillations in which the population is almost entirely controlled or automatic, can still occur in the local scenario, as shown by Fig. 3b which shows the average level of control over time in the entire local scenario. When the lag is sufficiently large, the population fluctuates between almost entirely controlled (<*x*> near 1) and almost entirely automatic (<*x*> near 0), as in the global case considered previously.

To quantify how globalization effects the relationship between lag and fluctuations, we examined the magnitude of the oscillations in controlled processing that emerged for local vs. global interactions at different levels of the lag parameter *τ*_*p*_. Specifically, we used the average value of the peak of *x* to quantify the size of the fluctuations. Figure 3c (see Supplementary Fig. 6 for results on other network structures) shows that, across different globalization conditions, increasing the lag *τ*_*p*_ leads to higher amplitude cycles (as in the well-mixed population^4^). However, when learning is local, a larger lag is required to produce cycles of equivalent magnitude. Thus, although local learning promotes spatial oscillations (as shown in Fig. 2), it suppresses aggregate-level temporal oscillations.

Why is this so? There are two contributing factors. First, the mesoscale communities created by local learning lead to decoupling, such that even if each section of the network is cycling with the same magnitude of the cycles observed under global learning, they are out of phase with each other. Thus, in aggregate, the cycles in the average level of control across the whole population are of smaller amplitude (see Supplementary Fig. 7). Second, when learning is local, each agent can only imitate a small fraction of the population; thus, it takes longer for the whole population to shift to the currently-optimal strategy. Hence, in order for a full population swing to occur (which causes the environment to move back in the other direction), the environment needs to stay put longer; that is, with local learning, it takes longer for strategic innovation to spread through the population, which mitigates the amplitude of the oscillations.

## Discussion

The simulation results reported above suggest that globalization can have a dramatic—and complex—impact on the evolutionary dynamics of cognitive control. They also reveal important distinctions between different forms of globalization. Although each form involves greater interaction with dissimilar others, the details of that interaction matter.

Consistent with most prior work on networks, we find that globalization of learning (i.e., being able to imitate successful others outside your local neighborhood) fundamentally alters the spatiotemporal dynamics of control, breaking up the mesoscale neighborhoods of individuals with similar cognitive strategies that exist when learning is local. Interestingly, a consequence of this homogenization is that global learning induces population-wide fluctuations in the level of control (because all agents are phase-locked).

The impact of the other forms of globalization, however, are specific to the particular system of automatic versus controlled processing we study here, and not obvious based on prior work. For example, the consequences of globalization of contact between agents depend on the nature of the contact. If automatic processing imposes costs on controlled processing (e.g., faster automatic agents outcompeting more thoughtful controlled agents when directly competing over resources, or automatic agents’ lack of planning undermining the plans of controlled agents), then globalization of contact harms control due to increased exposure to agents engaged in automatic processing. But if control benefits from interaction with automaticity (e.g., controlled agents selling products that are particularly helpful—and thus attractive—to automatic agents, or controlled agents exploiting the short-sightedness of automatic agents), then the opposite is true and globalization of contact promotes the evolution of control.

Finally, globalization of environment—that is, an increase in the extent to which one’s actions influence the environment experienced by everyone (such as the ozone layer)—uniformly reduces the level of control, by allowing automatic processing to enjoy the environmental benefits created by controlled processing. And surprisingly, it is global environment that facilitates the emergence of mesoscale communities and maintenance of variance in strategies within the population, whereas local environment leads to homogenization. This is the opposite of what one might expect from prior work on, for example, the evolution of cooperation^28^, in which (local) spatial structure maintains heterogeneity whereas (global) well-mixed populations are homogeneous. Together, these observations highlight the importance of the details when considering the impact of globalization—which form(s) of interaction are affected by globalization (learning, contact, and/or environment), and what underlying game structure is driving the dynamics (automatic/controlled processing, versus, for example, cooperation^45^).

In addition to these insights into the impact of globalization, our results also have implications for previous work on cognition-environment feedback^4–6^. Specifically, they lend support to the generality of prior conclusions about the emergence of cycles in automaticity versus control, which had previously been shown to occur when cognition-environment feedback is sufficiently lagged. First, they provide additional evidence for the robustness of such cycles, extending this observation to a broader and more complex set of conditions. More specifically, they show that under conditions of lagged feedback, cycles can occur in both local and global settings. Moreover, we find that when learning is local, spatiotemporal fluctuations can occur even in the absence of such lags. Thus, the current work strengthens formal theoretical support for the idea that societies characterized by high levels of cognitive control (and associated social and technological complexity) may be at risk of collapse due to cognition-environment feedback.

It is of course important to recognize that our model is, in the game theoretic tradition, an abstract and highly simplified conceptual model. Rather than quantitative predictions, our model makes high-level predictions about the kinds of outcomes that may arise, and the circumstances under which different outcomes might be expected to occur. These conceptual predictions can be explored in future work, using both laboratory experimental systems and examination of the observational data.

Finally, the insights generated by our model have the potential to help inform policy. Our results make several relevant points. First, they suggest that it may be of particular importance to promote interactions whereby control benefits from contact with automaticity, rather than suffers; such beneficial interactions make globalization of contact work for cognitive control instead of against it. For example, in a market context, facilitating the purchase by automatic agents of innovations created by controlled processing, the sale of which benefits the controlled agents (e.g., through marketing that appeals to automatic processing). Second, our results reaffirm the challenges posed by global-level environmental issues such as climate change, which create a free-rider problem that undermines cognitive control. And third, our results demonstrate the potential for a truly global collapse (and risk of extinction) of control when learning is global: as the whole world becomes bound together, there are fewer reservoirs of control to buffer against local fluctuations, and more cataclysmically, to repopulate after a large crash.

Globalization is an extremely complex issue, and it is essential to explore its causes and consequences using a wide range of perspectives and disciplines. We hope that the work presented here will add a new lens with which to consider the issue, provide new insights, and highlight important new directions for future work.

## Methods

### Agent-based model

Our simulation results are produced using agent-based simulations on a network with population size *N* = 100 (results are robust to larger population size; see Supplementary Fig. 4). Each agent *i* is defined by a single parameter *x*_*i*_ that represents its probability of controlled processing. The fitness of each agent is calculated according to the following function:

5 $$f_{i,t} = x_i( {1 - c + w ( {1 - < x > _{i,t}} )}) + ( {1 - x_i} )( {1 - p_{i,t}})$$

where *c* is the fixed cost of control, *w* is the impact of automatic processing on the cost of controlled processing, *<x>*_*i,t*_ is the average probability of control among the agent’s direct neighbors (including the agent itself) in generation *t*, and *p*_*i,t*_ is the relative advantage of controlled processing in generation *t*.

Agents update their strategies using the Moran death-birth process (our model is robust to the update rule, as additional simulations show that the pairwise comparison process^46,47^ leads to qualitatively similar outcomes). In each generation, an agent *L* (the learner) is randomly selected to potentially update its strategy. Then the learner is replaced by a copy of agent *T* (the teacher) who is selected with probability $W\left( {T \to L} \right) = \exp \left( {sf_T} \right)$ where *s* is the intensity of selection and *f*_*T*_ is the fitness of the teacher (reproducer). Alternatively, with probability *u* a mutation occurs; in that case, instead of adopting the other agent’s strategy, the learner adopts a new strategy sampled from a uniform distribution on the interval [0, 1].

Following ref. ^4^, we also implement cognition-environment feedback by updating the advantage of controlled processing in each generation with a time lag. When environment is local, we specify *N* agent-specific values *p*_*i,t*_ and stipulate that only the strategy of immediate neighbors of agent *i* (including agent *i* itself) influences the change in *p*_*i,t*_ over time:

6 $${{p}}_{{{i}},{{t}}} = {{p}}_{{{i}},{{t}} - 1} + \frac{{( {1 - < {{x}} > _{{{i}},{{t}} - 1}} ) - {{p}}_{{{i}},{{t}} - 1}}}{{\tau _{{p}}}}$$

Conversely, when environment is global, there is a single value of *p* for the whole population, which updates according to:

7 $${{p}}_{{t}} = {{p}}_{{{t}} - 1} + \frac{{\left( {1 - < {{x}} > _{{{t}} - 1}} \right) - {{p}}_{{{t}} - 1}}}{{\tau _{{p}}}}$$

*τ*_*p*_ represents the time lag of cognition-environment feedback. When *τ*_*p*_ = 1, the basic model described in the main text with no time lag recovers.

### Numerical simulations

Our simulations use intensity of selection *s* = 10 (strong selection) and mutation rate *u* = 0.01 (as expected, increasing the mutation rate pushes average values of *x* towards 0.5; see Supplementary Figs. 8 and 9). We structure the population within a ring in which each agent is connected to *k*/2 neighbors on each side (for a total of *k* neighbors). Simulations were initialized with *x* = 0.01 for all agents. We investigate the effect of globalization across 8 different scenarios described by the 2 by 3 combination of [local, global] × [learning, environment, and contact]. That is, for each dimension of globalization we either consider only neighboring agents (ring structure with *k* = 2; local) or the whole population (full graph; global). In a complimentary analysis (Supplementary Figs. 3, 5, and 6) we explored Small-world networks varying strength of community structure (rewiring rate).

We ran the simulations for 8 × 10^4^ generations except for the effect of *τ*_*p*_ on average peak of oscillations (Fig. 3) where we ran the simulation for 1.2 × 10^6^ generations. For *τ*_*p*_ = 1 where population reaches an equilibrium and stays there, we averaged over last 4 × 10^4^ generations for the stable strategy. Results reported in Fig. 1 and Fig. 3c are averaged over 10 simulation replicates. All simulations were done in parallel on MIT Sloan Engaging cluster.

### Reporting summary

Further information on research design is available in the Nature Research Reporting Summary linked to this article.

## Supplementary information

Supplementary Information

Reporting Summary

## Data Availability

All data required to run the simulations are available at: https://osf.io/fy94w/ or can be requested from the authors. A reporting summary for this Article is available as a Supplementary Information file.

## Code availability

All scripts necessary to reproduce the results are available at: https://osf.io/fy94w/ or can be requested from the authors.
