# Supplementary material for: Globalization and the rise and fall of cognitive control
Source: Nat Commun. 2020 Jun 18;11:3099. doi: 10.1038/s41467-020-16850-0 (PMC7303166; doi:10.1038/s41467-020-16850-0)
Supplement: Supplementary file 1 — Supplementary Information [file 41467_2020_16850_MOESM1_ESM.pdf]

Supplementary Information

"Globalization and the Rise and Fall of Cognitive Control"

Mosleh et al.

Sloan School of Management, Massachusetts Institute of Technology, Cambridge, MA 02138

E-mail: mmosleh@mit.edu

Contents

1. Supplementary Note 1: Analytical approximation for $\tau_p = 1$ 2

2. Supplementary Figures 4

2.1. Supplementary Figure 1. Variations of simulation outcomes for different replicates (local learning)........4

2.2. Supplementary Figure 2. Variations of simulation outcomes for different replicates (global learning)........5

2.3. Supplementary Figure 3. Aggregate-level controlled processing x for no lag $\tau_p = 1$ on Small-world network........6

2.4. Supplementary Figure 4. Aggregate-level controlled processing x for no lag $\tau_p = 1$ on a ring structure with N=10^5 nodes........7

2.5. Supplementary Figure 5. Meso-scale communities measures as a function of probability of link rewiring in Small-world network........8

2.6. Supplementary Figure 6. Effect of time-lag on amplitude of oscillation for Small-world networks varying probability of rewiring........9

2.7. Supplementary Figure 7. Local oscillation of neighboring agents with local learning ... 10

2.8. Supplementary Figure 8. Aggregate-level controlled processing x for no lag $\tau_p = 1$ and mutation u=0.2........11

2.9. Supplementary Figure 9. Aggregate-level controlled processing x for no lag $\tau_p = 1$ and mutation u=0........12

Supplementary Note 1: Analytical approximation for $\boldsymbol{\tau_p} = 1$

To give an intuition regarding the impact of globalization on the evolution of cognitive control in the absence of lag, we approximate the fitness of automatic and controlled processing for the simplified case in which an agent is either fully controlled or fully automatic (see Supplementary Table 1).

Supplementary Table 1. Approximation of fitness of automatic and controlled processing under different conditions of local versus global interaction and learning.

Environment	Contact	Learning	Fitness of controlled processing f_c	Fitness of automatic processing f_a
**Local**	**Local**	**Local**	$1 - \boldsymbol{c} + \frac{2}{3}\boldsymbol{\beta w}(1 - < \boldsymbol{x} >)(1 - \boldsymbol{a})$	$\frac{2}{3}\boldsymbol{\beta} < \boldsymbol{x} > (1 - \boldsymbol{a})$
Local	Local	Global	$1 - c + \frac{2}{3}w\beta(1 - < x >)$	$\frac{2}{3}\beta < x >$
Local	Global	Local	$1 - c + w(1 - < x >)$	$\frac{2}{3}\beta < x > (1 - a)$
Local	Global	Global	$1 - c + w(1 - < x >)$	$\frac{2}{3}\beta < x >$
Global	Local	Local	$1 - c + \frac{2}{3}w(1 - < x >)(1 - a)$	$< x >$
Global	Local	Global	$1 - c + \frac{2}{3}w(1 - < x >)$	$< x >$
Global	Global	Local	$1 - c + w(1 - < x >)$	$< x >$
Global	Global	Global	$1 - c + w(1 - < x >)$	$< x >$

First, we consider the impact of contact among agents, wherein the frequency of automatic processing impacts the payoff of controlled processing (via the $w(1 - < x >)$ term in f_c). When contact is global, the impact of contact is determined by the average level of automaticity in the population as a whole. When contact is local, the impact of contact is instead determined by the average level of control across the focal agent and her neighbors (of which there are two in the ring-structured population we study). Thus, the strategy of the focal agent has much greater impact on her contact payoff in the local case. In particular, if the focal agent is entirely controlled, then the average level of automaticity in her local neighborhood can be at most 2/3 (if both other agents were maximally automatic). As a result, the contact term in the payoff of controlled processing becomes $\frac{2}{3}w(1 - < x >)$ (as seen in rows 1, 2, 5 and 6 of Supplementary Table 1). This means that when automatic agents impose costs on controlled agents, $w < 0$, global contact will reduce the payoff of control relative to local contact (and thus reduce the equilibrium level of control). Conversely, when controlled agents benefit from contact with automatic agents, $w > 0$, globalizing contact will increase the payoff (and thus equilibrium level) of control.

Next, we consider environment, wherein the frequency of controlled processing determines the state of the environment and thereby the payoff of automatic processing ($f_a = < x >$). When

environment is global, all agents share the same environment described by the variable p, the value of which is determined by the average level of automaticity across the whole population. When environment is local, each agent experiences her own local environment that is determined by the average level of automaticity over her two neighbors and herself. Thus, as with contact, the focal agent's strategy has a much bigger impact on her local environment when environment is local compared to global. In particular, if the focal agent is fully automatic, then the average level of control in her local neighborhood can be at most 2/3 (if both other agents were maximally controlled). Furthermore, when environment is local, the focal agent also has a much larger impact on the environments experienced by – and therefore the optimal strategies of – her neighbors. If the focal agent is fully automatic, she decreases the p experienced by her neighbors, thereby creating selection pressure for them to become more automatic. Although the exact functional form of this impact is complex, we capture it conceptually by introducing a factor $\beta \leq 1$ that diminishes the level of control of an automatic agent's neighbors. Combining these two consequences of local interaction, the payoff of automatic processing becomes $\frac{2}{3}\beta < x >$ when contact is local (as seen in rows 1-4 of Supplementary Table 1). This means that regardless of model parameters, the payoff of automatic processing is lower (and therefore the frequency of control is higher) when contact is local relative to global.

These observations also imply a *synergy* between the locality of contact and environment, driven by local environment's impact on the optimal strategy of the focal agent's neighbors. As described in the preceding paragraph, local environment causes clustering of strategies (i.e., more automatic agents' neighbors to be more automatic and, symmetrically, more controlled agents' neighbors to become more controlled). When contact is also local, the neighbors which a controlled agent interacts with directly are more likely to be controlled by a factor β, leading to a further scaling of the w term in f_c (as seen in rows 1 and 2 of Supplementary Table 1). Thus, local environment amplifies the impact of contact on the average frequency of control (which, as described above, can be positive or negative depending on the sign of w).

Finally, we consider learning. When learning is global, no clustering or assortment in strategies arises due to the learning process. Thus (except for the assortment that arises from local environment captured by β), the average level of control among a focal agent's neighbors is equal to the population level of control $< x >$. When learning is local, however, this leads to assortment, such that a focal agent's neighbors are more likely than chance to have the same strategy as the focal agent. We follow common convention and define the level of assortment $\alpha \leq 1$, such that a controlled agent's neighbors will be controlled with probability $(1-\alpha) < x > +\alpha$, whereas an automatic agent's neighbors will be automatic with probability $(1-\alpha) < x >$. (As above, we forgo an exact calculation of the level of α, as the specific value of α does not qualitatively change its impact on the evolutionary outcomes). When both contact and environment are global, assortment (and thus local versus global learning) has no effect on the evolutionary outcomes: agents are no more likely to interact with their neighbors than any other agent, and thus the strategies of neighbors versus the population as a whole is irrelevant. When either or both contact/environment are local, however, local learning amplifies the other local effects: controlled agents' neighbors are more likely to be controlled by a factor α (appearing in the w term of f_c, lines 1 and 5 of Supplementary Table 1), and automatic agents' neighbors are more likely to be automatic by a factor α (appearing in f_a, lines 1 and 3).

Supplementary Figures

In this section, we provide numerical details of our simulation model and also show additional results for robustness of our result to Small-world networks varying probability of link rewiring.

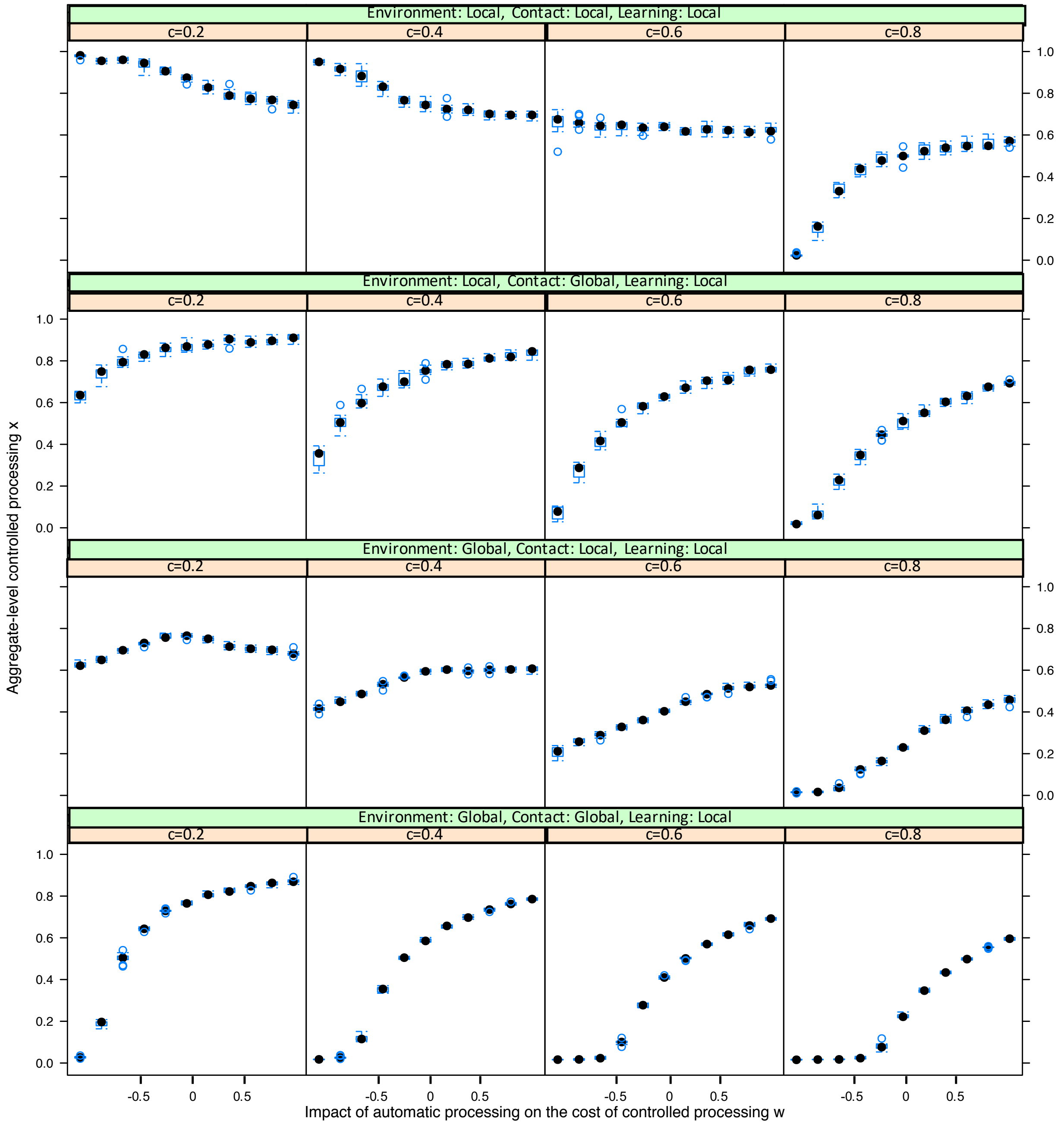

Supplementary Figure 1. Variations of simulation outcomes for different replicates (local learning). The boxplots in each panel shows variations of simulation outcome of aggregate-level controlled processing *x* for 10 replicates and for each given set of *w*, *c*, and localness.

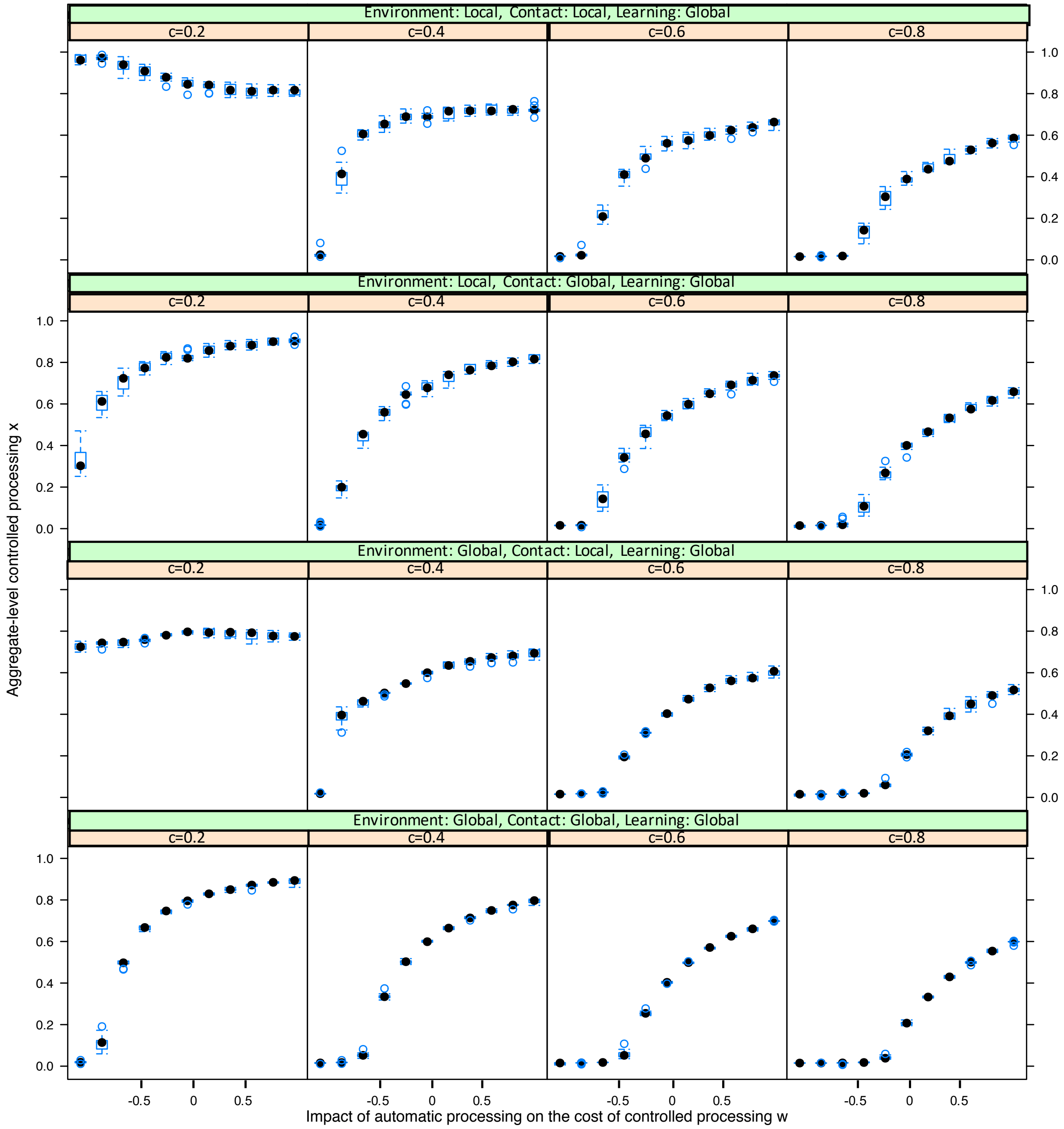

Supplementary Figure 2. Variations of simulation outcomes for different replicates (global learning). The boxplots in each panel shows variations of simulation outcome of aggregate-level controlled processing *x* for 10 replicates and for each given set of *w*, *c*, and localness.

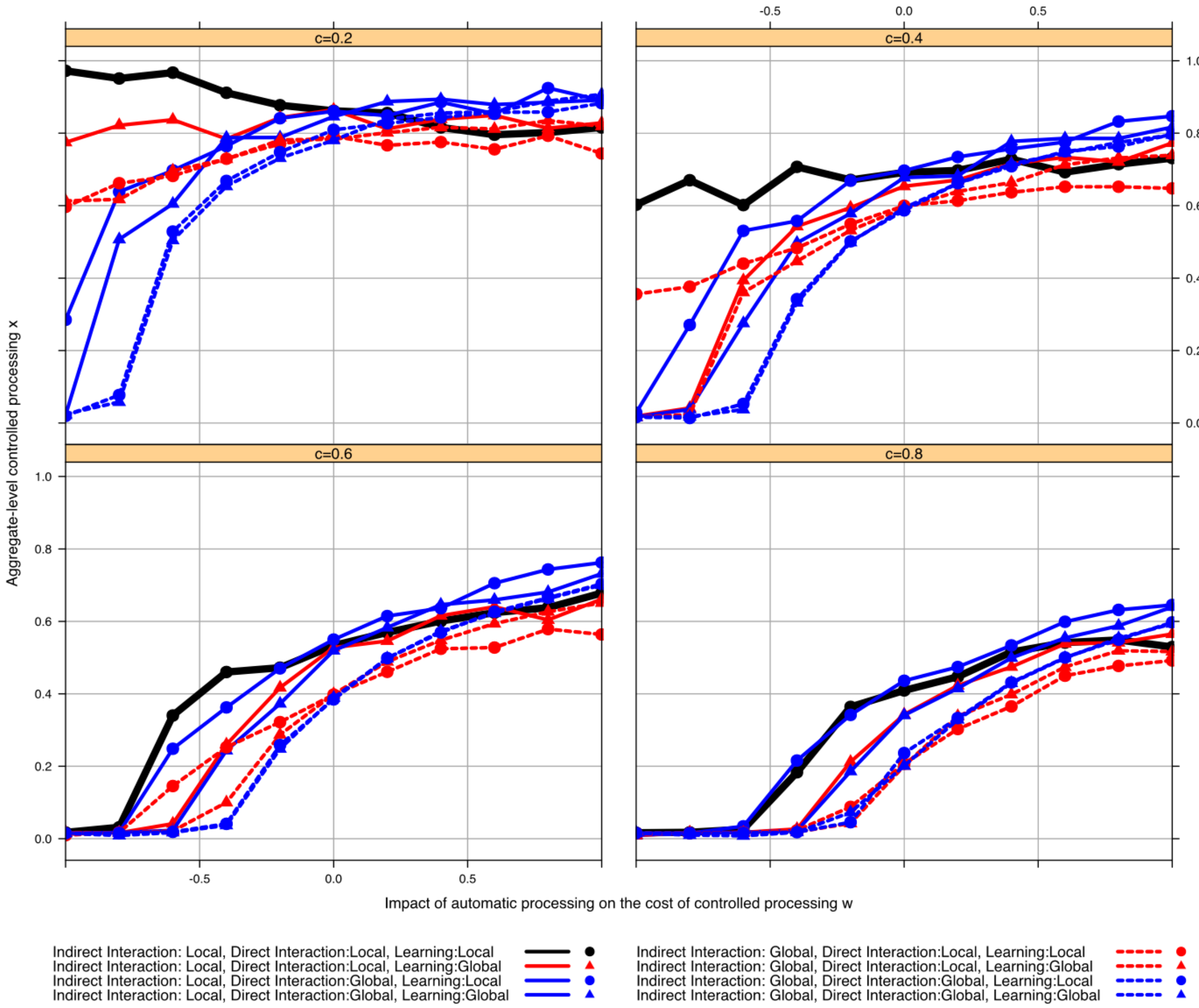

Supplementary Figure 3. Aggregate-level controlled processing x for no lag $\tau_p = 1$ on Small-world network where the average number of neighbors is 4 and probability of rewiring is 0.2. Each line represents a combination of local versus global contact, environment, and learning, across different levels of the impact of automatic processing on the cost of controlled processing (w) and fixed cost of controlled (c). Contact is indicated by color (red = local, blue = global). Environment is indicated by line type (solid = local, dashed = global). Learning is indicated by symbol (circle = local, triangle = global).

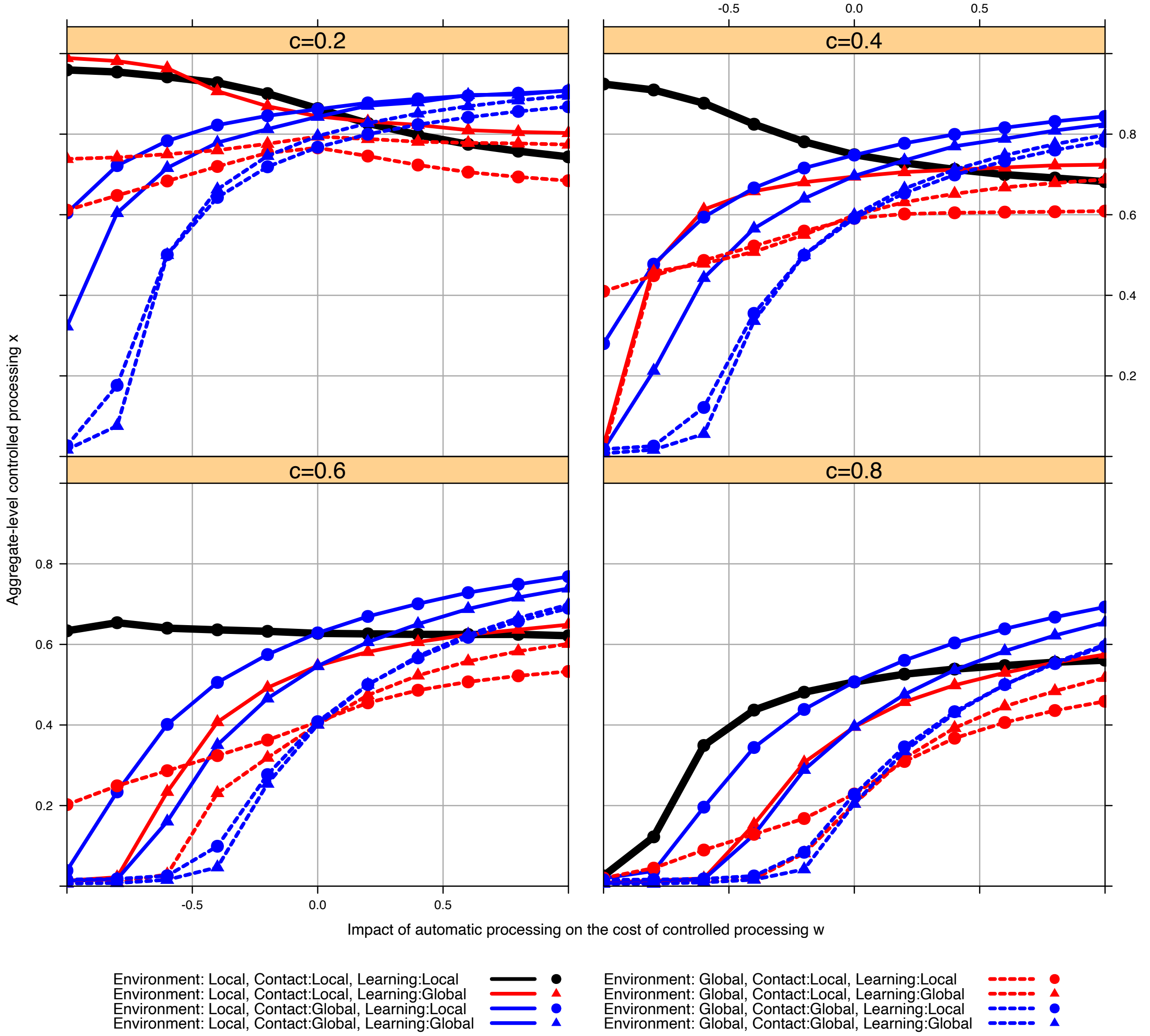

Supplementary Figure 4. Aggregate-level controlled processing x for no lag $\tau_p = 1$ on a ring structure with N=10^5 nodes where the average number of neighbors is 2. Each line represents a combination of local versus global contact, environment, and learning, across different levels of the impact of automatic processing on the cost of controlled processing (w) and fixed cost of controlled (c). Contact is indicated by color (red = local, blue = global). Environment is indicated by line type (solid = local, dashed = global). Learning is indicated by symbol (circle = local, triangle = global). Given the size of the network, the simulation was run for 3.6 $\times$ 10^7 generations to reach stable outcome for x.

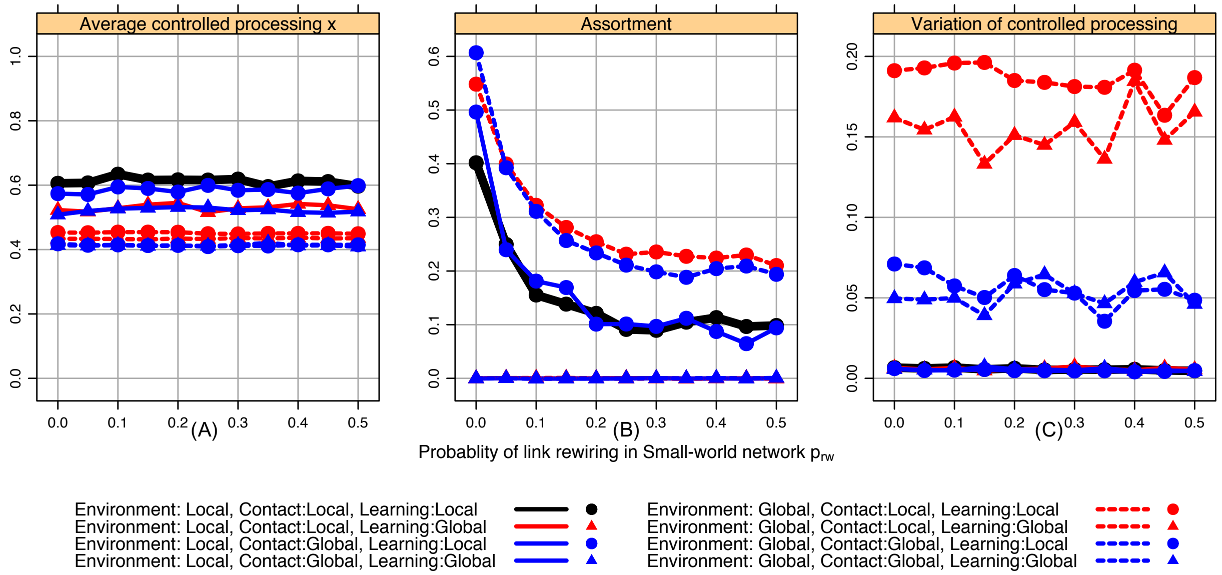

Supplementary Figure 5. Meso-scale communities measures as a function of probability of link rewiring in Small-world network. Each panel shows one measure averaged over last 4×10^4 generations for 10 simulation replicates. Results are generated on Watts-Strogatz Small-world networks, where each agent was connected to two other neighbors on each side (k=4), impact of automatic processing on the cost of controlled processing w=-0.15 and fixed cost of controlled c=0.5. All results of the ring structure qualitatively generalize to the Small-world networks. Aggregated controlled processing x and variation of strategies do not change with probability rewiring, yet assortment decreases as the probability of rewiring increases due to weaker community structure of the network.

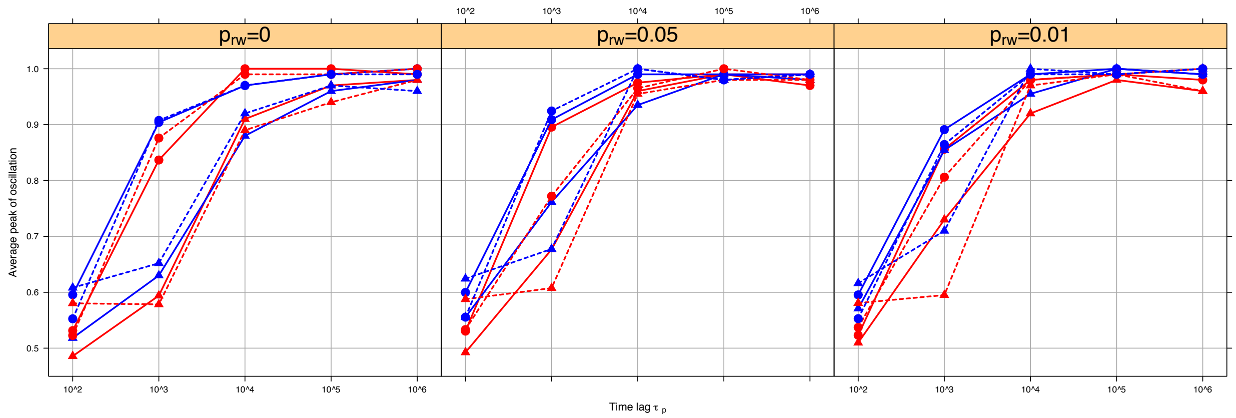

Supplementary Figure 6. Effect of time-lag on amplitude of oscillation for Small-world networks varying probability of rewiring *p_{rw}*. Increasing probability of rewiring weakens the effect of local learning on amplitude of oscillation as it decreases the strength of community structure in the network.

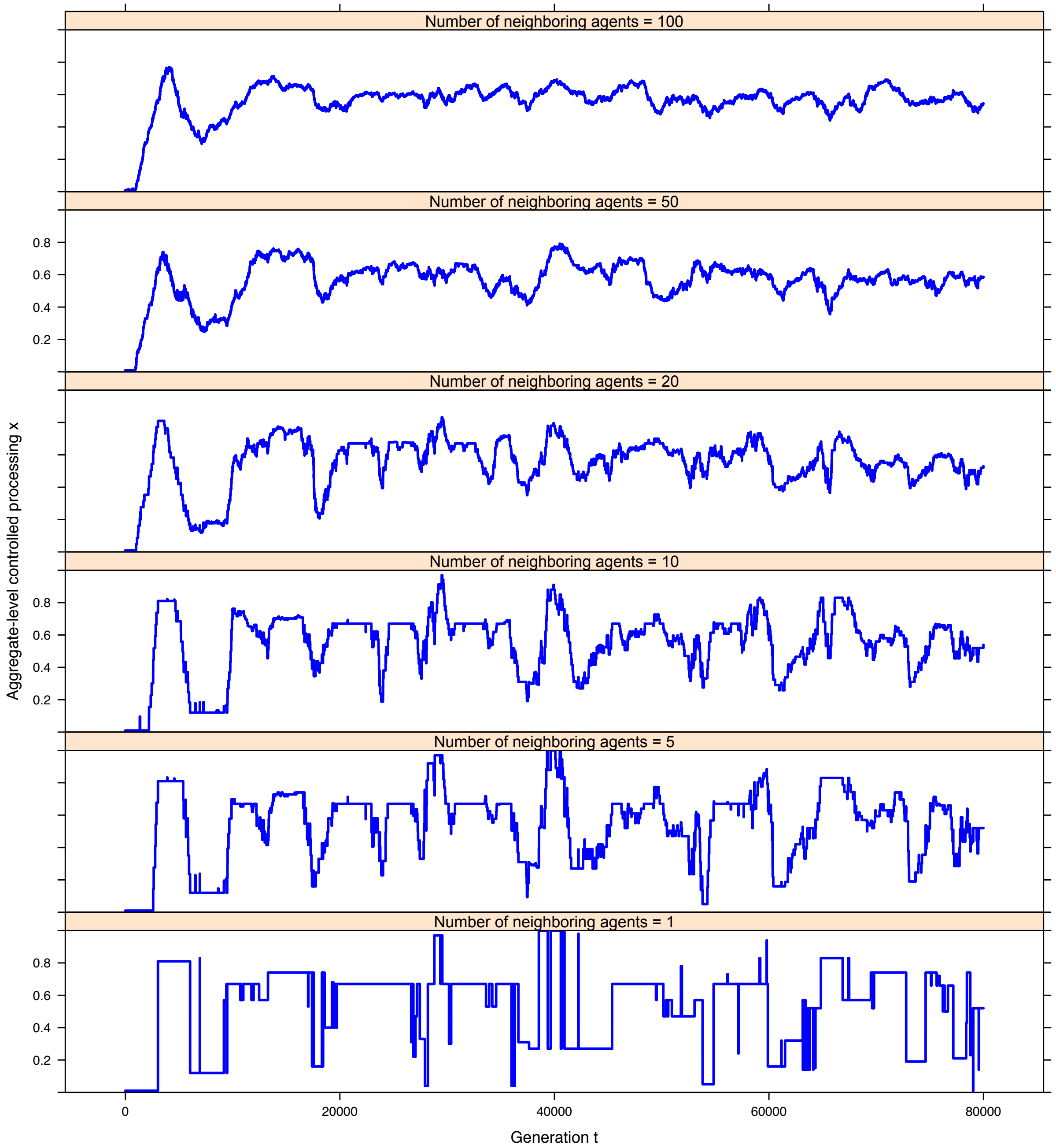

Supplementary Figure 7. Local oscillation of neighboring agents with local learning. Each panel shows oscillation of controlling processing *x* for a different number of agents located next to each other on a ring structure. Results are created for time lag $\boldsymbol{\tau_p} = 10^3$, the cost of controlled processing *w=-0.15*, fixed cost of controlled *c=0.5,* and local learning (the figure is generated with local environment and local contact; however, global environment and/or global contact do not qualitatively change these results).

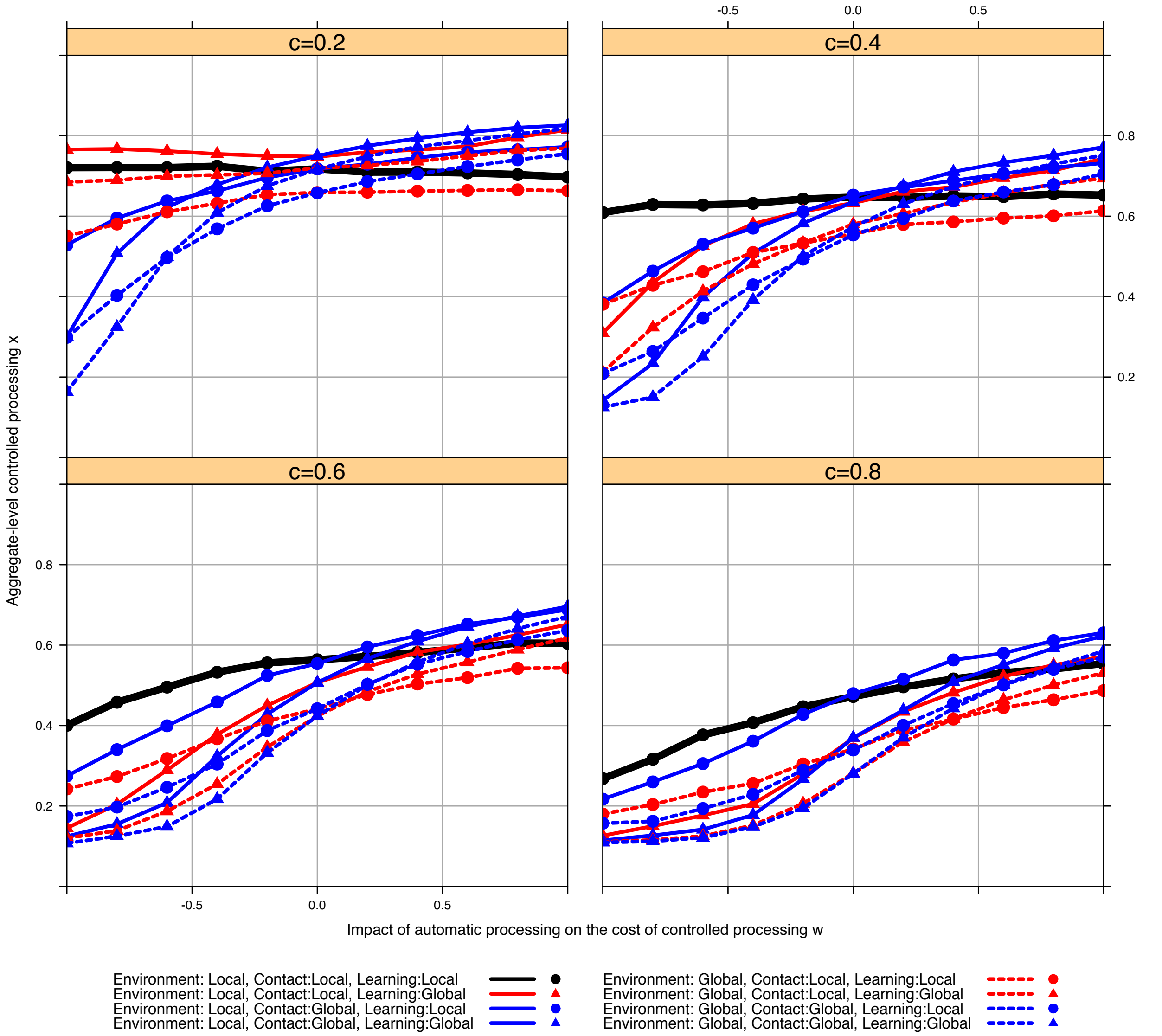

Supplementary Figure 8. Aggregate-level controlled processing *x* for no lag $\tau_p = 1$ and mutation *u=0.2*. Each line represents a combination of local versus global contact, environment, and learning, across different levels of the impact of automatic processing on the cost of controlled processing (*w*) and fixed cost of controlled (*c*). Contact is indicated by color (red = local, blue = global). Environment is indicated by line type (solid = local, dashed = global). Learning is indicated by symbol (circle = local, triangle = global).

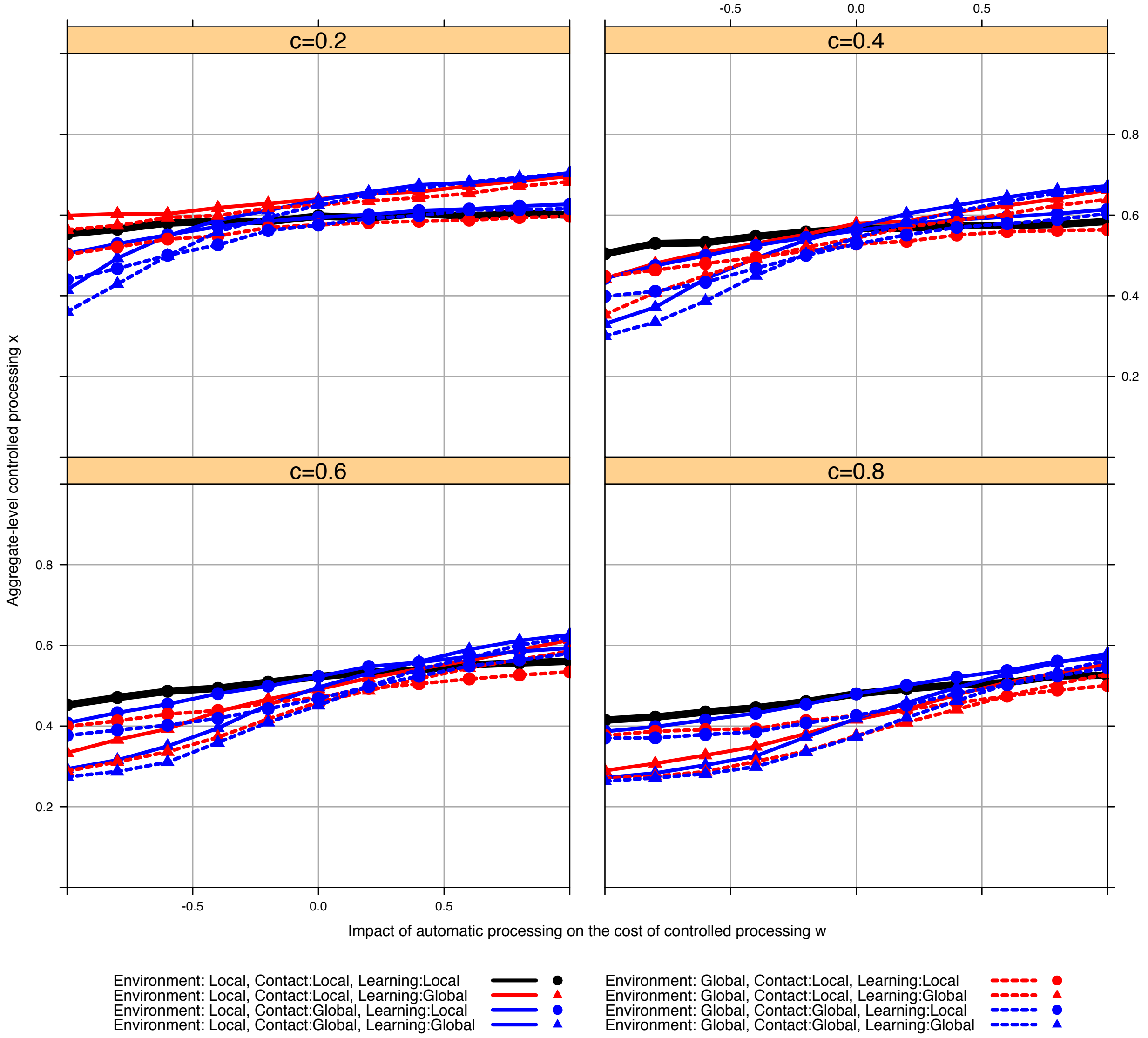

Supplementary Figure 9. Aggregate-level controlled processing *x* for no lag $\tau_p = 1$ and mutation *u=0.5*. Each line represents a combination of local versus global contact, environment, and learning, across different levels of the impact of automatic processing on the cost of controlled processing (*w*) and fixed cost of controlled (*c*). Contact is indicated by color (red = local, blue = global). Environment is indicated by line type (solid = local, dashed = global). Learning is indicated by symbol (circle = local, triangle = global).
